# Poisons and Poisoners

**Published:** 1892-06-11

**Authors:** 


### Poisons and Poisoners.

### THE MARQUISE DE BRINVILLIERS.

There is a certain interest attached to a great criminal, to one of those rare and terrible creatures who use death to gain an end with the calm temper with which most men perform their daily work. We can understand a blow given in sudden anger; we can even comprehend the slower burning hate that takes a murderous revenge; but beyond these there lies a class who kill without emotion, without hesitation, without remorse, when a human life stands as an obstacle in their path rather than deviate a hair's breadth from their chosen aim. They are the miracles of egotism, centring the universe in themselves, and therefore feeling themselves free to deal destruction with Olympian calm. They do not hate their victims, because they could never have loved them; they stand outside the range of human passions, and while their acts are diabolic they maintain in themselves an attitude that on the outside seems heroic. Of such a type was Marie Madeleine, Marquise de Brinvilliers, whose crimes have preserved her name to an evil immortality.

Her early history is that of the majority of Frenchwomen of rank in the eighteenth century. She was married when only eleven years of age to the Marquis de Brinvilliers, a selfish, careless, extravagant man, who neglected her entirely. Fear of his creditors prevented his coming to Paris often; he was more interested in his regiment than his wife, and the Marquise was left, so far as he was concerned, to go her own way. Perhaps it would have been better for her if his neglect had been even more complete. Once, when she was about eighteen, the Marquis came home for a visit of a few days, bringing with him an army friend, the Chevalier de Sainte-Croix.

The antecedents of Sainte-Croix were unknown; he himself took care to surround them with an air of mystery. But he was good-looking, well-mannered, and as attentive to the Marquise as her husband was indifferent; he attracted her, and when the Marquis lefc Paris, Sainte-Croix remained. Society at large took no heed of the relations of the Marquise and the Chevalier; to such things society in those days was used. But her own family, her father and brothers, were shocked and grieved at her conduct and made an effort to save her. To this end, however, they used evil means, and the result was only a lower depth of sin. They obtained a *lettre de cachet*, and Sainte-Croix was removed to the Bastille. There, the prison being over full, he was confined in the same cell as an Italian called Exili, who, having been driven out of his own country or his suspected complicity in various murders, had got into bad odour in Paris from a similar cause. The bodies of several of his supposed victims had been examined, but the poison, if any had been used, was so ingenious that it left no traces which the toxicology of that time could detect. Legally nothing could be proved against Exili, but the moral conviction of his guilt was so strong that he was imprisoned in the Bastille to prevent his further activity. Exili seems to have been a professional poisoner, to whom death-dealing assumed the proportions of an art. He would poison for the love of it, merely to test a drug; he was a sort of vivisector who pursued his experiments upon his fellow men, and felt scientific interest instead of human pity. Sainte-Croix became his willing pupil, and when at the end of a year he was set free, he carried Exili's secret with him.

It seems clear that after this Sainte-Croix lived by poisoning. He kept up a considerable establishment and spent freely, though he had no known source of income. Among his servants was a confidential servant called Martin, who was no other than Exili, who had been released from the Bastille not long after Sainte-Croix himself. But with the Chevalier's return to Paris, Madame de Brinvillier's family had taken up an attitude of vigilant protection, which she found embarrassing. Her father interfered against her associating with Sainte-Croix; therefore her father must be destroyed. Sainte-Croix gave her the means; and here comes in the first instance of the calm inhumanity of the woman. She tried the poison on her waiting-maid, and as the latter recovered from its effects she changed the drug for one of a more virulent character. Provided with this she set out with her father for Compiègne. At his country house there she mixed the poison with some broth, gave it him with her own hands, and was his apparently attentive nurse during the few days' illness that preceded his death. It was she who insisted on a physician being called in, and as the local doctor professed himself unable to explain the disease, insisted on taking the patient to Paris. But the Parisian practitioners were as much at fault as he of Compiègne. M. D'Aubray died, convinced that his daughter, whatever were her faults, was tender-hearted and affectionate.

But the crime was almost useless. Madame de Brinvilliers had counted on being free from a troublesome espionage, but her two brothers immediately assumed their father's place. She was in debt, and counted on her father's death adding to her income; but he bequeathed her only a small sum, and left the bulk of his property to his eldest son. Thus the knell of the brothers was rung. The Marquise was too shrewd in her vocation to use the same drug again; another, slower drug was wanted. About this time she developed a great appearance of charity. She visited the hospitals, and gave various dainties to the patients. The patients were soon after attacked by a slow, wasting fever, which no medicine seemed able to cure, and died within a month. Soon after Madame de Brinvilliers' brothers died with similar symptoms. Still no suspicion fell on her, and from this time she became more ready with her weapons. She attempted the life of her sister, she poisoned one of her own children, and took poison herself to test an antidote. So she says in her "Confession"—a sort of diary of her crimes which she kept—"for fear," Madame de Sevigné cynically suggests, "that these sins may escape her recollection." Still she held her own, except in so far as her intimacy with Sainte-Croix compromised her, and it was not until after his death that suspicion of her true character was aroused.

Sainte-Croix died in his laboratory. He was engaged in compounding one of those mysterious poisons of which we read in old chronicles, the very fumes of which are deadly. He wore a glass mask to protect himself, but by some accident the mask broke, and lacking its protection, he was poisoned by the fumes. Among his property was found a casket which contained various phials and powders, some of which were proved to contain arsenic and others antimony, while others defied analysis. This casket had been claimed by the Marquise, and she had shown such an anxiety to obtain it, that when its contents were known, there existed a firm conviction that she was a confederate of Sainte-Croix in his murderous work. She fled to Liège and took refuge in a convent. There she could not be arrested, but, being persuaded by a ruse to go outside the town, she was seized and brought to Paris. Among her papers was found the confession spoken of, which left no doubt of her guilt, though she continued for a time to deny it. At last she admitted her crimes, and it is after this that her character comes out in its most remarkable light. She was put to the torture in order to make her confess the names of her accomplices. Many have been known to make false accusations, in such a case, hoping thereby to have their punishment remitted. But Madame de Brinvilliers refused to compromise others, either with reason or without, and bore her torments with the utmost patience. She professed sincere repentance for her sins, and seemed moved to the deepest piety. We cannot help doubting the belated piety of that hard and merciless nature, yet it is undeniable that throughout the last scene of execution, with its long, cruel, and humiliating preliminaries, she behaved with the dignity and patience of a martyr. Was her conduct due to her newborn belief in the justice of God and hope of His mercy, enabling her to endure the punishment of her sins, or was it the outcome of her self-controlled nature, as powerful in restraining fear as pity? The mind hovers, uncertain and wondering, between the two hypotheses.

Her poisons seem to have been arsenic and antimony, and it is doubtful if in our day, with modern scientific knowledge, she would have escaped so long. As it is we know that suspicion, however vague, must have hovered round her long before the truth was brought to light, but she lived in an immoral society, where so many sins were condoned that no one regarded his neighbour's doings too closely. When the *lettre de cachet* can be obtained by private vengeance, the assassin with his more thorough *lettre de cachet* in the shape of poison and the knife will never be far behind. Madame de Brinvilliers, according to her confessor, had a balanced mind, singularly open to impressions of any kind; but the impressions offered her by society were all evil, and she became one of most unscrupulous criminals of her time.

